# Hypocortisolemic ASIA: a vaccine- and chronic infection-induced syndrome behind the origin of long COVID and myalgic encephalomyelitis

**DOI:** 10.3389/fimmu.2024.1422940

**Published:** 2024-07-09

**Authors:** Manuel Ruiz-Pablos, Bruno Paiva, Aintzane Zabaleta

**Affiliations:** ^1^ Faculty of Biological Sciences, Universidad Complutense de Madrid, Madrid, Spain; ^2^ Centro de Investigación Médica Aplicada (CIMA), IdiSNA, Instituto de Investigación Sanitaria de Navarra, Clinica Universidad de Navarra, Pamplona, Spain

**Keywords:** myalgic encephalomyelitis, long COVID, post-COVID-19 vaccine syndrome, ASIA syndrome, EBV, SARS-CoV-2, HLA-II, hypophysitis

## Abstract

Myalgic encephalomyelitis or chronic fatigue syndrome (ME/CFS), long COVID (LC) and post-COVID-19 vaccine syndrome show similarities in their pathophysiology and clinical manifestations. These disorders are related to viral or adjuvant persistence, immunological alterations, autoimmune diseases and hormonal imbalances. A developmental model is postulated that involves the interaction between immune hyperactivation, autoimmune hypophysitis or pituitary hypophysitis, and immune depletion. This process might begin with a deficient CD4 T-cell response to viral infections in genetically predisposed individuals (HLA-DRB1), followed by an uncontrolled immune response with CD8 T-cell hyperactivation and elevated antibody production, some of which may be directed against autoantigens, which can trigger autoimmune hypophysitis or direct damage to the pituitary, resulting in decreased production of pituitary hormones, such as ACTH. As the disease progresses, prolonged exposure to viral antigens can lead to exhaustion of the immune system, exacerbating symptoms and pathology. It is suggested that these disorders could be included in the autoimmune/adjuvant-induced inflammatory syndrome (ASIA) because of their similar clinical manifestations and possible relationship to genetic factors, such as polymorphisms in the HLA-DRB1 gene. In addition, it is proposed that treatment with antivirals, corticosteroids/ginseng, antioxidants, and metabolic precursors could improve symptoms by modulating the immune response, pituitary function, inflammation and oxidative stress. Therefore, the purpose of this review is to suggest a possible autoimmune origin against the adenohypophysis and a possible improvement of symptoms after treatment with corticosteroid replacement therapy.

## 1 Introduction

It is well recognized that various infectious diseases can generate dysfunction in the hypothalamus and/or pituitary gland, affecting its normal functioning (1–9). There are cases of hypothalamic-pituitary insufficiency due to intracellular pathogens such as herpes simplex type I and II, cytomegalovirus, Epstein-Barr virus (EBV), enterovirus, influenza A virus, varicella zoster, Coxsackie B virus, human immunodeficiency virus (HIV), hantavirus Puumala, Mycobacterium tuberculosis, Borrelia burgdorferi, Treponema pallidum, Toxoplasma gondii and Candida (1–15). These infections can cause direct damage to the tissues of the hypothalamus and pituitary gland due to inflammation and lesion formation, which can result in alterations in the production and regulation of key hormones for the homeostasis of the organism.

The vulnerability of the adenohypophysis to infection by intracellular pathogens is largely due to the lack of an effective blood-brain barrier in the pituitary (16–18). The absence of this barrier allows these infectious agents to enter directly into pituitary tissue, where they can trigger a localized inflammatory response and cause direct damage to endocrine cells (15, 19, 20). In addition, these pathogens can take advantage of various strategies to evade or suppress the host immune response, which facilitates infection and viral persistence in the adenohypophysis (20). Involvement of the adenohypophysis by these pathogens can lead to the dysfunction of hypothalamic-pituitary-adrenal (HPA) axis, resulting in hormonal dysregulation and a wide range of clinical manifestations (15, 19).

Cortisol exerts several innate and adaptive immunosuppressive effects. These include inhibition of NF-κB activity, suppression of T-cell function through the interaction with the interferon-gamma (IFN-γ) and tumor necrosis factor-alpha (TNF-α) pathways, alteration of blood cellular composition by decreasing lymphocytes and increasing myeloid cells, and inhibition of antigen presentation by specialized cells (21, 22). For this reason, hypercotisolemia induces an immunosuppressive state that predisposes the patient to various bacterial, viral, fungal and parasitic infections (22). On the other hand, hypocortisolism is associated with an increased likelihood of developing inflammatory and autoimmune diseases (23). Under normal conditions, cortisol helps to regulate the inflammatory response, but low cortisol levels reduce the inhibition of the nuclear factor kappa B (NF-κB) pathway and T lymphocyte activation, leading to a proinflammatory environment (proinflammatory cytokines such as IFN-γ) and immune hyperactivation (23–25). Therefore, under conditions of hyperactivation of the immune system due to interaction with antigens from pathogens or adjuvants in individuals with genetic predisposition (HLA-II), the development of hypopituitarism could allow maintaining the state of immune hyperactivation and subsequent immune exhaustion.

## 2 Hypopituitarism in the long COVID

Leow et al. observed that approximately 40% of patients with severe acute respiratory syndrome (SARS) associated with SARS-CoV infection may develop reversible secondary adrenal insufficiency, suggesting possible pituitary inflammation in these patients (26). Wei et al. (27) observed reduced immunostaining of corticotropic, somatotropic, and thyrotropic cells in SARS-CoV-infected patients. In addition, given the high genetic similarity between SARS-CoV and SARS-CoV-2, adenohypophysitis has also been observed during the acute phase of COVID-19, with secondary adrenal insufficiency being higher in patients with long COVID (LC) (20, 28–35).

Three mutually non-exclusive mechanisms have been proposed that could alter the HPA axis. Direct infection of pituitary or adrenal tissue, direct damage to the adrenal gland or pituitary caused by the immune system due to antibody production and/or cell damage, and inhibition of the HPA axis induced by inflammatory cytokines (functional hypopituarism) (36).

Supportive of direct infection, angiotensin-converting enzyme 2 (ACE2) is the receptor used by SARS-CoV2 to infect cells and is expressed in a large number of tissues, including the adenohypophysis (20, 37, 38). The ability of SARS-CoV-2 virus to induce adrenal insufficiency has been supported by the presence of ACE2 receptors in both the adrenal gland and adenohypophysis (20, 36–38). This could explain the direct damage in some of these patients and the alteration of the HPA axis (20).

Regarding direct damage due to the immune system, it has been suggested that there is a high molecular similarity between SARS-CoV-2 peptides and human adrenocorticotropic hormone, as in the case of SARS-CoV viral peptides that are structurally similar to adrenocorticotropic hormone (ACTH) (36, 39, 40). The recognition of these viral proteins induces antibody production, which result in collateral destruction of host ACTH, causing adrenal insufficiency in individuals carrying certain haplotypes of human leukocyte antigen (HLA) system genes (36, 39). Although it is yet to be found which peptide has ACTH mimicry, it has been observed that critically ill patients with COVID-19 have elevated titers of anti-ACTH antibodies, suggesting a possible explanation for the adrenocorticotropic impairment that is associated with SARS-CoV-2 infections (40, 41). To accomplish this, the virus produces amino acid sequences that mimic host ACTH (42). When the host produces antibodies against these viral antigens, the antibodies also bind to ACTH, limiting the host stress response by decreasing the ability to stimulate corticosteroid secretion (42). In addition, the strategy of directing its host immune system against the host itself by molecular mimicry would allow the virus to better survive during a poorly directed and weakened immune response (42).

Finally, in terms of inhibition induced by inflammatory cytokines, chronic exposure to proinflammatory cytokines such as TNF-α, could suppress pituitary ACTH secretion and thus, cause relative hypocortisolism during chronic infections (36, 43–46). This cytokine dysregulation strategy is also shared by other viruses, such as influenza and SARS-CoV, where they induce an increased release of proinflammatory cytokines to alter the immune response and inhibit the host anti-inflammatory response through the production of ACTH autoantibodies (42). Thus, this increase in proinflammatory cytokines could also have an indirect effect on the pituitary by decreasing ACTH secretion.

## 3 Hypopituitarism in myalgic encephalomyelitis

Something similar could happen in patients with myalgic encephalomyelitis or chronic fatigue syndrome (ME/CFS), where lower ACTH levels at 6 months after diagnosis of EBV induced infectious mononucleosis were highly predictive of the development of ME/CFS (13). It has also been observed that fatigue and pain are associated with cortisol levels in saliva, being more severe in those ME/CFS patients with lower cortisol levels (47–51). In addition, different studies have shown a loss of the morning ACTH peak (52) and reduced ACTH response in the insulin challenge test (53).

In the study by Steiner et al. it was observed that the onset of ME/CFS was frequently related to an acute respiratory infection or suspected viral infection (54). Approximately 19% of the patients were found to have experienced a primary EBV infection during adolescence, which is a well-known trigger of ME/CFS (54). Furthermore, it was highlighted that a primary EBV infection in adulthood is considered a risk factor for various autoimmune diseases (55).

In this case, within the stellate follicle cell population of the adenohypophysis there is a subpopulation of pituitary dendritic cells, which play a role in communicating immune activation to the HPA axis and express HLA-II, and may be a target of EBV infection in some patients (56, 57). These pituitary dendritic cells facilitate communication between the immune and endocrine systems by regulating ACTH secretion in response to infections through the release of proinflammatory cytokines, such as IL-6 (57). In addition, these cells have also been shown to be involved in mediating the negative feedback of glucocorticoids on ACTH release (57). Thus, direct EBV infection of these cells could generate alteration of the HPA axis by decreasing ACTH secretion and generating hypocortisolism.

Patients with ME/CFS may also present autoimmune hypophysitis (58). Bellis et al. observed a high prevalence of anti-hypothalamic antibodies, and anti-pituitary antibodies in patients with ME/CFS, showing a possible correlation between pituitary dysfunction (low ACTH levels) and the presence of these antibodies in high titers (59). Furthermore, it suggests that hypothalamic-pituitary autoimmunity may be triggered by infectious events, as occurs in LC, and lead to hormonal deficiencies (59). The presence of anti-hypothalamic antibodies, and anti-pituitary antibodies is not only observed in patients with idiopathic adenohypophysis deficiency, they can also be found in some patients with previously normal pituitary function who subsequently developed pituitary hormone dysfunctions (59, 60). This suggests that severe symptoms in ME/CFS, such as orthostatic hypotension and severe sleep disturbances, might be related to impaired pituitary function due to autoimmune damage, whereas moderate/mild symptoms might be related to less pituitary damage (59).

## 4 Post-vaccine hypopituarism

In 2011, Shoenfeld et al. presented the concept of adjuvant-induced autoimmune/inflammatory syndrome (ASIA), which describes a series of immune-mediated disorders that can be developed in genetically predisposed individuals (61, 62). Patients with these diseases present similar clinical features, which usually improve when the triggering factor is removed (63). The development of these pathologies requires the presence of a favorable genetic background (61), where adjuvants might induce an exacerbated immune response causing adverse reactions without necessarily involving resolution of pathological condition (20).

A classic example of ASIA is the autoimmune complications of immune checkpoint inhibitor (ICI) therapy used in cancer treatment, known as immune-related adverse events (irAEs) (61, 64). In contrast to traditional autoimmune diseases, irAEs are usually not chronic (64). In this case, the “adjuvants” are monoclonal antibodies that inhibit cytotoxic T lymphocyte antigen 4 (anti-CTLA4), programmed cell death-1 (anti-PD-1) or its ligand PD-L1 (anti-PD-L1), enhancing antitumor immune responses (19, 61). These ICI can remove the immunological brakes, resulting in the activation of anti-tumor CD8 and CD4 T cells (19, 61). This overstimulation of the immune response can break self-tolerance and lead to autoimmune reactions in different organs, as ICIs inhibit inhibitory signals that prevent T-cell activation against self-antigens (19, 61, 65, 66). One of the main autoimmune disorders associated with this therapy is the development of autoimmune hypophysitis and isolated adrenocorticotropic hormone (ACTH) deficiency (19, 67).

The main side effects of ICI therapy may include chronic fatigue, unrefreshing sleep, sleep disturbances, cognitive problems and memory loss in up to 42% of cases (68). It is believed that these effects may be associated with immune hyperactivation in the central nervous system (CNS) (68), along with endocrine disturbances such as hypophysitis (69). These endocrine disturbances result in decreased cortisol synthesis, which in turn may prevent adequate inhibition of T-lymphocyte activation (70, 71).

Vaccine-triggered autoimmune syndromes fall within the scope of the ASIA syndrome (20). It is believed that adjuvants such as aluminum salts, emulsions, oils, spike (S) protein, Toll-like receptor agonists, mRNA vaccine lipids, and polyethylene glycol could promote immune hyperactivation with autoimmunity in genetically susceptible individuals (HLA-DRB1) (20, 36, 62, 72–75). Although information on SARS-CoV-2 vaccine-related endocrine complications is limited, some cases of hypophysitis have been observed (20, 33, 62, 76–81).

Although there are still no data on which adjuvants are involved in the development of autoimmune hypophysitis after vaccination against SARS-CoV-2, we suggest that vaccines using viral vectors could play an important role. Some of the SARS-CoV-2 vaccines use adenoviruses as vectors (82, 83). Adenoviruses are linear, double-stranded DNA viruses and remain in an episomal condition in the nucleus (84). The broad ability of adenoviruses to infect a variety of tissues and stimulate a strong immune response makes them one of the most effective viral vectors for inducing a robust immune response against the target antigen (84, 85). There are four SARS-CoV-2 vaccines based on adenovirus vectors, AstraZeneca’s vaccine (AZD1222) which uses a replication-deficient chimpanzee adenovirus vector genetically modified to carry the SARS-CoV-2 protein S gene, the CanSino Biologics vaccine (Ad5-nCoV), the Janssen-Johnson&Johnson vaccine (Ad26Cov2-S), and the Gamaleya Research Institute vaccine (Sputnik V) that use other adenoviruses as vectors carrying SARS-CoV-2 protein S genome (82–84). These vaccines are similarly designed, using adenovirus vectors with substitution of the early adenoviral gene (E1) for the SARS-Cov-2 S gene in the adenoviral DNA and additional deletion of E3 (82, 83). It has been observed in animal studies that adenovirus DNA vector without a replicating E1 gene can remain transcriptionally active in the body for months after vaccination (86) whereas in the case of mRNA vaccines, the RNA is rapidly degraded (87, 88). Persistence in antigen expression could differentiate vaccines employing adenovirus vectors from the rest, and this feature is thought to favor the generation of long-term immune responses and prolonged immunity (82, 85). It has been observed that adenoviral vectors that persist in the body are highly immunogenic and are responsible for strong CD8 T cellular and/or humoral immune responses specific to the transgenic antigen (85, 86). In addition, the chronic expression of the vector protein S gene, in individuals carrying certain HLA haplotypes, could induce immune hyperactivation and excessive production of proinflammatory cytokines, leading to autoimmune hypophysitis or inhibition of ACTH secretion (functional hypopituitarism) (20, 62). There is evidence that adenoviral vector vaccines may induce higher levels of specific T cells compared to mRNA vaccines (82, 89), which may increase the likelihood of autoimmune cell hyperactivation in genetically susceptible individuals. Therefore, all these characteristics make these vectors a potential risk for the development of autoimmune diseases, including autoimmune hypophysitis, in genetically predisposed individuals (HLA-DRB1), as occurs in ASIA syndrome (20, 62, 77, 80). However, it should not be ruled out that SARS-CoV-2 mRNA vaccines could also generate autoimmune responses in genetically susceptible individuals upon exposure to viral antigens, as observed in some cases (76–81), but as the mRNA degrades and does not persist in the organism (88), the long-term autoimmune sequelae could be limited and disappear once the triggering antigen is removed, as occurs in many cases of ASIA syndrome (64, 74)

## 5 Similarity in genetic predisposition

Single nucleotide polymorphisms (SNPs) in several genes associated with autoimmune diseases have been observed in patients with ME/CFS (54). Significant associations were identified between the SNPs PTPN22 rs2476601 and CTLA4 rs3087243 and ME/CFS, especially in patients who reported acute onset of disease after infection (54). This suggests that autoimmunity may play a role in infection-triggered ME/CFS, and highlights the relevance of genes that regulate B- and T-cell activation in this disease (54).

In addition, there is evidence that patients with ME/CFS have associations between elevated fatigue and specific SNPs in TNF-α, IL1b, IL4, and IL6 genes (90). It has also been observed that ME/CFS shares a number of SNPs in HLA, IFN-γ, 5-HT, and NR3C1 genes with other disease-related fatigues (90). Especially, HLA class II genes were found to be associated with ME/CFS (91–94). This has led several research groups to look for an association between the expression of certain HLA-II alleles and the development of ME/CFS. Accordingly, HLA-DQA1*01 (91), HLA-DQB1*06 (91), DQB1*0303 (92), HLA-DQ3, HALA-DR4, and HLA-DR5 (93) have been associated with an increased risk to develope ME/CFS, but the strength of the data supporting this association is limited (93, 94). One of the limitations of these studies is that they did not differentiate between the infectious-onset vs noninfectious-onset of ME/CFS, as did Steiner et al. (54). This relationship provides further evidence that an autoimmune mechanism may be involved, given that the relationship between certain HLA-II alleles and autoimmune diseases is widely recognized (95). In addition, HLA-DR genes and their products have been found to play a role in the regulation of the immune system, both in resistance and susceptibility to different infectious agents (90, 96). Importantly, polymorphisms in HLA-DR genes have been associated with other diseases in patients with chronic fatigue, such as chronic Q fever (97). Therefore, current information on immune alteration and cytokines in ME/CFS suggests that the HLA system may play a role in the regulation of the immune system and its effectiveness in fighting certain infections and may cause the symptom of chronic fatigue (90, 91, 96, 98).

It is also important to note that an association with several SNPs in the glucocorticoid receptor gene, nuclear receptor subfamily 3 group C member 1 (NR3C1), has been identified in ME/CFS (54, 99). This receptor influences the activity of the HPA axis by regulating the inflammatory response through the release of cortisol (99, 100).

The development of ASIA involves exposure to adjuvant stimuli (such as silicone implants, drugs, infections, metals, or vaccines) in genetically susceptible individuals, especially those with variants in genes such as HLA-DRB1 and PTPN22 (61). This exposure leads to excessive activation of the immune system and production of autoantibodies, eventually resulting in autoimmune diseases (61). Certain genes, such as HLA-II genes, have been found to be associated with both autoimmune diseases and ASIA syndrome (96, 101). In addition, the tendency for autoimmune diseases to run in families suggests a common genetic basis (61, 96). The prevalence of certain alleles such as HLA-Cw12, HLA-DR15, HLA-DQ7 and HLA-DPw9 was significantly higher in patients with isolated ACTH deficiency due to ICI treatments, whereas the presence of HLA-Cw12 and HLA-DR15 was significantly higher in patients who developed ICI-induced hypophysitis (102). In addition, it has been observed that genetic polymorphisms of CTLA-4 and PD-1 genes may also increase the incidence of immunotherapy-induced hypophysitis and other autoimmune diseases (19). In the case of post-vaccine subtypes of ASIA syndrome, such as human papillomavirus (HPV), SARS-CoV-2 and rubella vaccines, a higher incidence is observed in genetically predisposed individuals with HLA-DRB1 alleles, the most frequent being HLA-DR15 (74, 96, 103, 104).

The main haplotype able to recognize with high affinity the greatest number of protein S-derived peptides, and thus, trigger the strongest cellular and humoral response after vaccination against SARS-CoV-2, is the HLA-DRB1*15:01~DQA1*01:02~DQB1*06:02 (105–109). Although the HLA-DRB1*15:01 allele appears to be associated with a more potent immune response against SARS-CoV-2 peptides (105–109), this does not necessarily indicate a better antiviral response. For example, Novelli et al. observed that DRB1*15:01, -DQB1*06:02, and -B*27:07 alleles predispose patients to a worse course of COVID-19 disease (110, 111). Thus, this excessive response associated with this allele may be more closely related to an increased risk of autoimmunity (112). The fact that the HLA-DRB1*15:01 allele presents a greater number of peptides with high affinity suggests that it has an intrinsic ability to bind and present a wide range of peptides derived from the virus S protein and thus to activate a greater number of T and B cells (105–109). This observation is consistent with the DRB1*15:01 allele belonging to one of the three oldest ancestral haplotypes (DR2-DQ6, DR3-DQ2, and DR4-DQ8), which have survived over time due to their ability to recognize a greater diversity of antigens, allowing them to detect and eliminate a greater number of pathogens (113, 114). The ability of these HLA class II alleles to recognize a greater number of antigens and to stimulate a greater number of T and B cells could increase the probability of activation of autoreactive T cells due to their promiscuous nature (112). This, in turn, could lead to cross-reactions in individuals carrying these haplotypes after exposure to foreign antigens, as occurs during infections or vaccinations. Thus, if the ability to recognize and present a high range of peptides is coupled with a higher cross-reactive capacity of the HLA-DRB1*15:01 allele compared to other alleles, this could increase the risk of developing autoimmunity, rather than providing effective protection against the virus (112). Interestingly, data from a computational analysis suggest that the HLA-DRB1*15:01 allele may present high affinity for autoepitopes after vaccination with BNT-162b2 mRNA due to cross-reactivity, the induced immune response may neutralize the function of several proteins or lead to direct tissue damage, manifesting fatigue, pain and other neurological symptoms (115). This same vaccine has been associated with isolated cases of hypophysitis and isolated ACTH deficiency (76, 80, 81).

Lio et al. (116) observed that individuals with HLA-DR2 (HLA-DR15 and HLA-DR16) showed significantly increased antiviral antibody titers (herpes simplex type 1, cytomegalovirus, EBV and rubella virus) compared to individuals with other HLA-DR types. In studies using DR1(*0101)/DR2(*1501) heterozygous human T-cell lines, a significant decrease in the *in vitro* T-cell response to rubella virus was observed when the antigen was presented by HLA-DR2-expressing monocytes compared with HLA-DR1-positive monocytes (104, 117, 118). In addition, a lower T-cell response to pathogenic antigens has been documented in HLA-DR2-positive individuals (119, 120). Zdimerova et al. showed in their study that, upon infection of mice engrafted with human immune cells carrying HLA-DR15+ or HLA-DR15- (DR4), EBV viral load and CD8 T cell expansion were higher in those HLA-DR15+ mice, and myelin basic protein-specific CD4 T cells were only detected in HLA-DR15+ mice (121, 122). Despite a hyperactivation of CD8 T cells, EBV viral loads were less controlled in HLA-DR15+ mice (121, 122). Specifically, CD4 T-cell clones showed lower efficiency in recognizing EBV-transformed B-cell lines expressing HLA-DR15+ (121, 122). Thus, HLA-DR15 may result in impaired activation of the HLA-II-mediated CD4 T response, increased viral load/EBV latency cells, increased levels of anti-EBV nuclear antigen 1 (anti-EBNA-1) antibodies, and expansion of CD8 T cells, which may be less effective inducing viral clearance (121, 122). Since B cells with EBV type I latency are only controlled by EBNA-1-specific CD4 T cells (123–125), these results suggest a reduction in the immune response to EBV infection in HLA-DR15+ individuals, while proinflammatory effects, as well as an increased CD4 T-cell cross-reactivity may increase the risk of developing autoimmune diseases, such as multiple sclerosis (121, 122).

Therefore, HLA-DR2 could have a negative effect on the antiviral T cell responses different pathogens, preventing control of the initial viremia and leading to increased viral persistence and higher antibody titers (104, 121, 122).

## 6 Common model of acquired immunodeficiency and hypocortisolism

**Figure 1:**
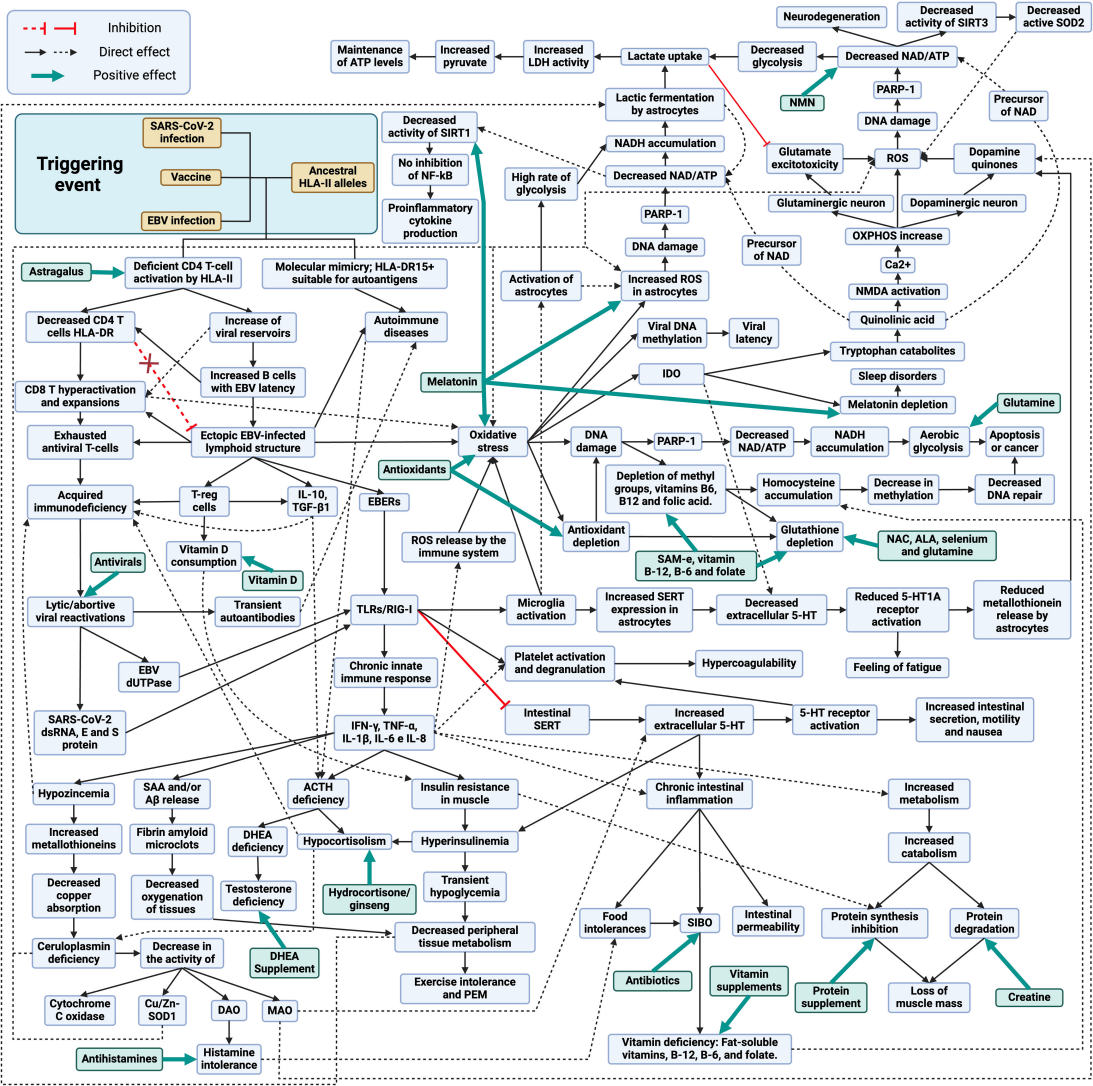
Schematic model of the development and treatment of long COVID, post-COVID-19 vaccine syndrome and myalgic encephalomyelitis/chronic fatigue syndrome. Created with BioRender.com.

Interaction with foreign antigens and adjuvants upon infection or vaccination in individuals with ancestral HLA-II alleles, such as HLA-DR15 (**Figure 1**), results in deficient HLA-II-mediated CD4 T-cell presentation and activation (61, 90, 93, 94, 96, 98, 106, 121, 122). In the case of viral infections or vectors, this could result in an increase in viral reservoirs due to a defective immune control (104, 121, 122). In response to this increase in viral reservoirs, there would be a hyperactivation and expansion of CD8 T cells and an increase in antibody levels against foreign antigens (e.g., increased EBNA-1 IgGs) (85, 86, 121, 122). On the other hand, deficiency in CD4 T-cell activation results in decreased immune control of other latent pathogens such as herpesviruses or parvovirus B19 (126–128). Either by EBV primoinfection or infection by another pathogen that decreases CD4 T-cell responsiveness would increase the number of EBV-latent cells in different tissues, as EBV type I latent B cells are only controlled by EBNA-1-specific CD4 T cells (123–125). The escape of these cells from immunosurveillance results in the formation of ectopic EBV latency lymphoid structures in different tissues that promote inflammatory responses by releasing EBERs, producing abortive reactivation and releasing new virions (129–133). Increased viral reactivation may increase the production of transient autoantibodies and the release of viral dUTPases (129, 134). In addition, increased foreign viral antigens from EBV reservoirs, SARS-CoV-2 or vaccine viral vectors, in individuals carrying ancestral HLA class II alleles with the ability to detect a greater number of antigens and activate a greater number of T and B cells, could increase the activation of autoreactive T cells due to their promiscuity and molecular mimicry of self-antigens with viral antigens (112). This would give rise to cross-reactions with self-antigens generating autoimmune diseases such as autoimmune hypophysitis (112). Finally, an immunological exhaustion of T cells (PD-1+/Tim-3+) is produced by chronic exposure to viral antigens, with consolidation of the disease (135, 136).

The increase of viral reservoirs and EBV latent cells trigger different evasion mechanisms, such as the deficient cytotoxic response of viral specific CD4 T cells through the release of IL-10, recruitment of regulatory T cells (Tregs) and release of miRNA contained in exosomes (129). Activation of TLR3 by EBERs or TLR2 by EBV dUTPases results in the release of proinflammatory cytokines (IL-1β, IL-6, IL-8, IL-12, TNF-α, and IFN-γ) and IL-10 through the activation of NF-κB (137, 138). In the case of SARS-CoV-2, genomic ssRNA activates TLR7/TLR8, dsRNA intermediates during viral replication activate TLR3, and viral E and S proteins activate TLR-2 (139–141). Therefore, in both cases, chronic activation of innate immunity due to viral persistence could occur (142).

Elevated levels of IL-6, IL-10 and TNF-α and increased expression of immune checkpoint markers such as PD-1 and T-cell immunoglobulin mucin-3 (Tim-3) on the surface of peripheral T cells, reduce T-cell function and memory T-cell activity (143, 144). Persistently increased proinflammatory cytokines can lead to impaired immune responses (145). A clear example of this relationship can be observed after the administration of tocilizumab, an IL-6 receptor antagonist that restores the number and cytotoxic activity of CD4 and CD8 T lymphocytes (145).

The persistent chronic inflammation, induced by the production of proinflammatory cytokines, increases the demand for vitamin D by the immune system (146). A key role of vitamin D is to promote immune tolerance, which is achieved in part through the promotion of Tregs cells, which help to keep the immune equilibrium and prevent autoimmunity (147). For this reason, in inflammatory diseases, vitamin D intake by immune cells tends to increase, which can lead to vitamin D deficiency (146), contributing to insulin resistance (148)

Hypozincemia may occur due to increased proinflammatory cytokines, as they increase metallothionein expression, leading to a reduction of zinc availability in blood and its uptake by immune cells (149, 150). The increase of metallothioneins in enterocytes reduces copper (Cu) uptake and ceruloplasmin synthesis (150–152). Decreased Cu transport leads to decreased activity of Cu-dependent enzymes (CU/Zn-SOD, DAO, MAO and cytochrome C oxidase) (152–154). The decrease in diamine oxidase (DAO) activity would alter histamine metabolism, which could trigger the onset of symptoms due to its accumulation (155). On the other hand, the lack of monoamine oxidase (MAO) activity, enzymes involved in the oxidative deamination of mitochondrial monoaminergic neurotransmitters such as dopamine, serotonin, tyramine, and norepinephrine, would also result in the accumulation of these neurotransmitters (154).

In addition, the increase in proinflammatory cytokines leads to the formation of fibrin amyloid microclot resistant to fibrinolysis, either by increased production of serum amyloid A (SAA) or by the release of β-amyloid (Aβ) peptide by platelets, resulting in capillary obstruction (156–159). High levels of IFN-γ produced by NK cells in response to persistent infection cause insulin resistance by down-regulating insulin receptor transcription in myocytes (160, 161). To compensate for insulin resistance, the pancreas increases insulin production, leading to compensatory hyperinsulinemia (160–162). Hyperinsulinemia reduces glycogenolysis in the liver, resulting in transient hypoglycemia and decreased metabolism in peripheral tissues, with subsequent exercise intolerance (162).

Autoimmune hypophysitis or elevated TNF-α levels could suppress ACTH secretion in the pituitary and, together with chronic exposure to IL-10, TGF-β1 and TNF-α and hyperinsulinemia could also suppress ACTH-stimulated cortisol secretion in the adrenal gland (43, 44, 163–169). Low cortisol levels could also influence ceruloplasmin levels, since activation of glucocorticoid receptors increases ceruloplasmin expression (170, 171).

It is also important to mention that sex hormones may play a role in the severity of symptoms. Since testosterone is immunosuppressive, it has been suggested that low levels led to an increase in autoimmune diseases by not suppressing autoreactive T cells (104, 172). Lack of ACTH reduces the production of dehydroepiandrosterone (DHEA) and dehydroepiandrosterone sulfate (DHEA-S) in the adrenal glands in both men and women, leading to lower testosterone levels in both sexes (173, 174). Although men may produce testosterone through the luteinizing hormone (LH) pathway in the testes (175), this production may be lower than in healthy individuals due to the reduction of available DHEA and may favor inflammatory responses. In addition, it has been observed that DHEA levels can influence the immune response to chronic infections by promoting Th1 responses and inhibiting IL-6 production (176, 177). Consequently, decreasing its levels could also influence the deficient cellular response to chronic infections.

During chronic infections, increased levels of proinflammatory cytokines such as TNF-α, IFN-γ and IL-6 together with increased CD8 T cell activation can trigger muscle catabolism and even cachexia, promoting the degradation of muscle proteins for energy or amino acids (178–180). This process is activated upon increased demand for these nutrients and could facilitate a rapid increase in nutrient accessibility to fuel adaptive immune responses and inflammatory processes (180).

TLR2 and TLR3 activation also causes inhibition and decreased expression of intestinal 5-HT transporters (SERTs), resulting in extracellular accumulation of 5-HT and activation of 5-HT receptors, generating increased motility, nausea and intestinal secretions (181–184). The intestinal serotonergic alteration together with the increase of proinflammatory cytokines also generates chronic inflammation, resulting in decreased acid secretion, breakdown of the intestinal barrier, decreased expression of enzymes necessary to digest carbohydrates, and alteration of the intestinal adaptive immune system, leading to the appearance of food intolerances and bacterial overgrowth in the small intestine (129, 181, 182, 184–187). The onset of SIBO causes malabsorption of fat-soluble vitamins, vitamin B-12, B-6 and folate (186). When activated through 5-HT2A receptors by increased 5-HT in peripheral blood or by binding of EBERs to TLR3 or by activation of TLRs by SARS-CoV-2, platelets bind fibrinogen, leading to increased aggregation and clotting processes (188–190).

Viral exosomes, EBER, protein S and SARS-CoV-2 intermediate dsRNAs activate TLRs receptors on endothelial cells of the blood-brain barrier, triggering the release of proinflammatory cytokines and altering barrier integrity (191–193). Both infected cells crossing the compromised blood-brain barrier and virions and exosomes containing viral genetic material can activate microglia via TLR3 and TLR2 receptors (191, 194).

The increase in both proinflammatory cytokines and oxidative and nitrosative stress by activation of microglia through TLRs could lead to increased IDO activity in microglia, resulting in reduced tryptophan levels, increased quinurenine catabolites, and decreased 5-HT and melatonin synthesis (195, 196). Quinolinic acid (quinurenine metabolites) is a precursor of nicotinamide adenine dinucleotide (NAD) that has neurotoxic properties through its binding to the postsynaptic N-methyl-D-aspartate (NMDA) receptor, followed by a sustained influx of Ca2+ leading to increased oxidative and nitrosative stress (197, 198). The increase in neuronal overexcitation via NMDA generates an increase in Ca2+ entry into the cytosol that is buffered by mitochondria (197). One of the consequences of excessive Ca2+ uptake by mitochondria is that mitochondrial respiration and mitochondrial membrane potential increase, leading to an increased likelihood of electron leakage and reactive oxygen species (ROS) production (197). That is, quinolinic acid may have toxic effects on the CNS, contribute to oxidative stress and neuroinflammation in both dopaminergic and glutaminergic neurons (198). In addition, quinolinic acid activates the NMDA glutamate receptor in the presynaptic neuron and stimulates the release of glutamate or dopamine, depending on the type of neuron, generating greater accumulation of neurotransmitters (129, 199). On the other hand, the activation of astrocytes by the increase of proinflammatory cytokines causes an increase of intracellular ROS that decreases the expression of glutamate transporters and increases their release, thus increasing the levels of extracellular glutamate and generating more neurotoxicity by a greater activation of NMDA receptors (199).

In this situation of neuronal oxidative stress with chronic activation of microglia, a process known as the astrocyte-neuron L-lactate shuttle (analogous to the reverse Warburg effect) is triggered (200–202). This cycle involves a crucial interaction between astrocytes and neurons to maintain ATP levels and energy homeostasis (200). In response to oxidative stress, neurons undergo overactivation of the enzyme poly (ADP-ribose) polymerase-1 (PARP1) to prevent DNA damage, which decreases intracellular NAD and ATP levels, compromising the efficiency of glycolysis and oxidative phosphorylation, affecting the ability of neurons to generate ATP (200, 203). In parallel, astrocytes increase their metabolism, glycolysis and lactate fermentation to produce lactate (204). In such circumstances, diverting metabolism to lactic fermentation is beneficial, as this anaerobic process produces less ROS compared with mitochondrial oxidative phosphorylation (205). This lactate produced is released into the extracellular space and taken up by nearby neurons, where it is converted back to pyruvate and enters directly into the citric acid cycle in the mitochondria, generating ATP through oxidative phosphorylation (200, 204). The utilization of lactate as an energy source allows neurons to maintain their ATP levels even when aerobic glycolysis is compromised by NAD depletion (200). This mechanism is essential to overcome oxidative stress and maintain neuronal function under these proinflammatory conditions (200). Furthermore, both astrocytes and neurons can use microglia-derived quinolinic acid as an alternative source to replenish NAD stores (206, 207).

Thus, if neuroinflammation remains over time and the astrocyte-neuron L-lactate shuttle cannot cope with chronic oxidative stress, this would result in chronic PARP-1 activation with decreased NAD levels within the cell (198, 202). This reduction in NAD decreases ATP production and leads to defects in DNA repair, as NAD is essential for PARP-1 activity, which increases the accumulation of genetic damage and potentially contributes to the development of neurodegenerative diseases (198, 208). Eventually, this may lead to cell dysfunction and death (208). Likewise, NAD depletion contributes to oxidative stress, as NAD enhances antioxidant capacity by increasing glutathione levels and antioxidant enzyme activity (209). Furthermore, a reduction in NAD levels would result in decreased activity of sirtuins, such as SIRT3 (mitochondrial sirtuin), leading to less deacetylation and, consequently, less activation of SOD2 (210). This in turn would lead to decreased ROS neutralization and increased intracellular oxidative stress (210). Reduced NAD also reduces SIRT1 activity, which limits its ability to inhibit the NF-kB pathway (211). This perpetuates the inflammatory response by increasing the release of proinflammatory cytokines in microglia cells and astrocytes (211).

On the other hand, TLR3 receptor activation in microglia leads to increased release of IL-1β and TNF-α, which increases SERT expression in astrocytes and thus 5-HT reuptake and its degradation to the metabolite 5-HIAA, thereby reducing extracellular 5-HT levels (212–216). The decrease in 5-HT reduces neuronal 5-HT1A receptor activation (213–215, 217). In addition, 5-HT1A receptor activation is also involved in ACTH hormone secretion (218). Therefore, a decrease in serotonin availability in the brain could also result in decreased 5-HT1A activation and decreased ACTH synthesis. Normally, when serotonin binds to 5-HT1A receptors, it can inhibit glutamate release, helping to maintain a balance of this neurotransmitter (219). However, if serotonin levels decrease, there may be less activation of 5-HT1A receptors, which may contribute to symptoms such as fatigue and the development of depression (213–215, 217). In addition, these 5-HT1A receptors are also found on astrocyte cells in the brain (220). When astrocytes detect activation of these receptors, they may respond by producing and releasing metallothioneins into the extracellular space (220). Therefore, if there is a decrease in serotonin levels due to an increase in serotonin reuptake, and consequently a lower activation of 5-HT1A receptors, astrocytes may produce less metallothioneins. This could reduce the protection that these proteins provide to dopaminergic neurons, increasing their vulnerability to oxidative stress damage (129, 220). Consequently, a decrease in Cu availability in these cells due to a decrease in metallothionein production and Cu uptake in the intestine would result in a decrease in dopamine degradation by the MAO pathway, since the activity of this enzyme is Cu-dependent (129, 154). If MAOs cannot efficiently break down dopamine due to lack of Cu, more dopamine may accumulate in the cytosol of neurons and may undergo autoxidation (221). Then, under conditions of Cu deficiency in dopaminergic neurons, there might be an increased propensity for the formation of dopamine quinones and ROS due to autoxidation of accumulated dopamine (129).

Another contributor to the chronic state of oxidative stress in the body is the increase in CD8-depleted T cells (135, 136). The metabolic programs of T cells undergo significant changes during their activation and depletion (136). Naïve T cells rely primarily on oxidative phosphorylation to generate ATP, whereas activated T cells shift their metabolism toward aerobic glycolysis (Warburg effect) to meet the demands of growth, proliferation, and effector functions, such as IFN-γ production (136). However, continued hyperactivation can lead to uncontrolled IFN-γ production and contribute to the development of autoimmune diseases (222). In addition, prolonged antigen exposure and continued stimulation of CD8 T cells leads to CD8 T cell exhaustion, which represents a significant challenge to the effectiveness of the immune response against chronic viral infections and tumor development (135, 136, 223). Exhausted T cells show a reduced capacity to perform metabolic processes such as aerobic glycolysis and oxidative phosphorylation (OXPHOS), both due to external influences and intrinsic signals, such as PD-1 expression (136). This decrease in metabolic capacity, together with changes in signaling cascades and epigenetic landscapes, leads to mitochondrial dysfunction and increased ROS production (136). These changes weaken the effector function of T cells and impair their immune responsiveness, including the production of cytokines such as IFN-γ, resulting in compromised responsiveness in exhausted T cells (136).

Increased oxidative stress in the body can cause DNA damage in various cells due to increased ROS, which in turn can lead to increased PARP-1 activity to activate DNA repair (203). This increased activity can result in NAD depletion and NADH accumulation (203). In response, these cells may shift their metabolism toward aerobic glycolysis, generating lactate as an end product, to reduce ROS production by avoiding oxidative phosphorylation, and to regenerate NAD through lactic fermentation (205). This increase in plasma lactate is essential for activated T cells and also supports the increased lactate demand by neurons due to increased oxidative stress (200). However, if chronic oxidative stress persists due to infection, it could lead to apoptosis of these cells or the development of mutations and cancer (224). On the other hand, increased oxidative stress can increase DNA methylation (hypermethylation), leading to increased utilization of methyl groups (225, 226). These metabolic processes require the presence of vitamins B-12, B-6 and folate (225). However, both increased utilization of these vitamins for methylation and decreased intestinal absorption due to inflammation can alter the homocysteine cycle (225, 227, 228). This alteration can lead to a decrease in the availability of methyl groups for methylation (hypomethylation), resulting in an accumulation of homocysteine in the blood (225, 228).

Elevated PARP-1 levels not only help against DNA damage, but are also exploited by EBV, as PARP1 acts as a barrier to productive lytic reactivation (229) and promotes the transition of EBV into the LMP1-induced latency state (230). Moreover, elevated DNA methylation could be exploited by EBV-infected cells for transformation through viral DNA methylation, preventing their replication and favoring the establishment of different types of latency that allow evasion of the immune system (226).

## 7 Treatment

Given the occurrence of immune exhaustion in these viral infection-related syndromes, one might think that the use of ICI, such as anti-PD1 and anti-CTLA4 antibodies, could be helpful in reactivating the immune system, but these antibodies may not be beneficial in this specific context (231). It has been observed that their administration could exacerbate the inflammatory response by increasing the levels of proinflammatory cytokines, which play a role in the development of these pathologies (231). Moreover, this therapy could exacerbate pre-existing hypophysitis or autoimmune diseases and further worsen the condition of affected patients (19, 232). Therefore, ICI could reactivate exhausted T cells and worsen preexisting autoimmune hypophysitis (19), further increasing subsequent T-cell exhaustion by elevating the level of inflammatory cytokines (231, 233). Surprisingly, CTLA-4 blockade has been observed to increase the expression and activity of the enzyme indolamine 2,3-dioxygenase (IDO), which could further contribute to immune exhaustion (234). Similar process has been observed during chronic infections such as that caused by HIV/AIDS, where IDO enzyme was activated in tissues of infected hosts (234). IDO converts tryptophan to quinurenine, which stops T-cell proliferation and activation (234–236). This process is induced by the interaction between CTLA-4 expressed on Tregs or activated T cells and CD80 or CD86 molecules on the surface of antigen presenting cells (APCs) (234). Although this IDO activation is considered an immune response, it can impair the effectiveness of the immune response, since in excess it limits the availability of tryptophan, an amino acid essential for T-cell function (235, 237). During the immune exhaustion characteristic of chronic infection, an increase in CTLA-4 expression is observed, which may increase IDO activation (234). Surprisingly, blockade of CTLA-4 results in a significant increase in IDO expression and activity (234). It is believed that increased immune activation induced by CTLA-4 blockade may trigger a compensatory response that leads to an increased IDO expression, further contributing to immune exhaustion (234). Therefore, the presence of immune exhaustion in infectious syndromes could be a compensatory mechanism to slow-down the progression of chronic infection, as occurs with AIDS patients (234, 236, 238). This is because IDO activation could prevent the spread of intracellular pathogens by inhibiting their replication by depriving them of tryptophan (236). However, by not controlling infection, this increase in IDO activity is exploited by the host to prevent excessive tissue destruction, despite the fact that this leads to inhibition of antiviral T-cell responses, expansion of Tregs and tolerance to infection (196). All this means that treatment with ICI represents a possible increased risk of worsening in these patients, by increasing possible baseline hypophysitis, development of autoimmunity, inflammation and viral reactivations of latent pathogens (239–241). In particular, the use of anti-CTLA4 antibodies has been associated with the development of autoimmune hypophysitis, where only a small number of patients may discontinue glucocorticoid replacement after ICI therapy due to irreversible pituitary damage (67).

On the other hand, evidence suggests that a short course of corticosteroids at the onset of the disease may be beneficial in patients with autoimmune hypophysitis after viral infections or vaccinations and may improve the associated immunological alterations (20, 26, 35, 74, 79, 242–244). It is believed that corticoids-mediated inhibition of ACTH secretion reduces the production of anti-ACHT antibodies, which in turn allows an increase in the anti-viral humoral response against the viral antigen with molecular mimicry with human ACTH (42). Altogether, these data suggest that by eliminating auto-reactive humoral response against ACTH and by compensating for the lack of ACTH, the immune response against infection is improved (42). By eliminating the ACTH-evasive strategy of ACTH mimicry presented by these pathogens, it favors a more efficient immune response (42). For this reason, corticosteroid treatment should be performed as soon as possible, since it allows the use of lower doses of corticosteroids and in a shorter period of time, which can minimize the adverse effects associated with high and prolonged doses (42). When administered late upon infection, higher doses and longer treatment periods may be required due to the presence of overactive T-lymphocyte responses and uncontrolled secretion of proinflammatory cytokines, which might result in irreparable damage (42). If corticosteroid treatment is prematurely discontinued, before the infection has been completely cleared, symptoms may reappear again as viral antigen with ACTH mimicry remains (42). This is where the combination of antivirals with a short course of corticosteroids (**Figure 1**) could accelerate viral clearance, in cases that have been initiated by infection (243). By reducing viral replication in the early stages of the disease, it facilitates the immune system to resolve the infection more quickly and decreases the production of viral antigens that could mimic autoantigens in genetically predisposed individuals (243). In addition, the use of antivirals could also decrease T-cell exhaustion by decreasing chronic exposure to viral antigens. If the infection and inflammation resolve after treatment and the pituitary parenchyma is not destroyed, remission could occur (35, 245, 246). However, it is crucial to keep in mind that if, at the end of corticosteroid and antiviral treatment, symptoms reappear with a decrease in ACTH levels, this could indicate potentially permanent pituitary damage (pituitary atrophy) (78, 245, 246). In such a case, chronic administration of replacement doses of corticosteroids may need to be contemplated to preserve adrenal function (76, 78, 245, 246).

Treatment may be useful in classic cases of ASIA syndrome or non-latent infections with little pituitary damage, where corticosteroids can be used temporarily until complete elimination of the adjuvant or virus (35, 243, 244, 246–248). However, complications arise when the damage is permanent, treated late, or when the adjuvant (e.g., vaccines) or latent viral infection (e.g., herpesvirus) chronically persists in the body, directly (persistence of ACTH-mimetic viral antigen) or indirectly affecting the HPA axis (via proinflammatory cytokines) (10, 67, 76, 246, 249). In such cases, chronic replacement therapy with corticosteroids could be considered, albeit with the risk of pituitary and adrenal atrophy (246, 250). Conversely, if there is no evidence of pituitary damage and ACTH levels are normal, but there is a hyporesponsiveness of the HPA axis, it would be prudent to consider other treatment options before resorting to prolonged corticosteroid use. For example, the use of supplements that reduce inflammation, oxidative stress, and stimulate cortisol production, such as ginseng (251, 252).

Ginseng could act by reducing the NF-κB pathway, which in turn decreases the production of proinflammatory cytokines such as TNF-α, IL-6, and IL-1, which could be inhibiting ACTH and cortisol secretion (43–46, 252, 253). This mechanism would result in increased cortisol levels, which in turn would reduce IFN-γ production and improve patients’ symptoms (43–46, 252, 253). In addition, inhibition of the NF-κB pathway mediated by ginseng allows decreased microglia-mediated neuroinflammation and PD-L1 expression, improving the imunological response and decreasing immune depletion (251, 252, 254).

**Figure 2:**
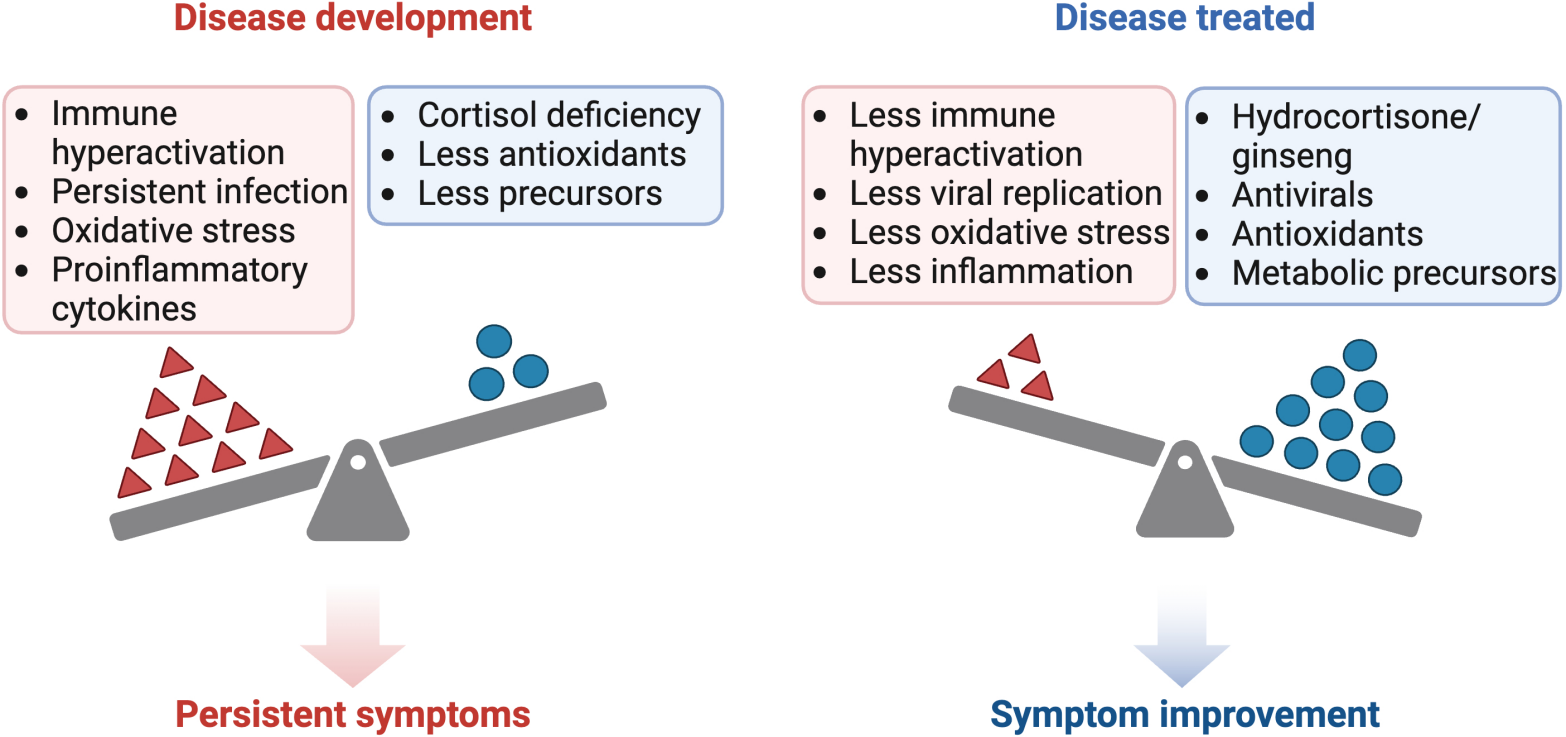
Comparison of the pathogenesis and treatment of ME/CFS and long COVID. Created with BioRender.com.

Both with the use of corticosteroids and with the use of ginseng, it would be necessary to consider supplementation with DHEA, since the use of corticosteroids inhibits ACTH secretion and therefore the production of DHEA and DHEA-S (174, 255). To optimize the therapeutic approach in inflammatory and immunosuppressive states, it is proposed to complement conventional therapy with antivirals and corticosteroids/ginseng with the administration of supplements that mitigate oxidative stress and replenish precursors depleted during inflammation or by malabsorption phenomena (**Figure 2**). These supplements include antioxidants such as vitamin C, as well as compounds that help to restore glutathione levels in the body, such as n-acetylcysteine (NAC), alpha-lipoic acid (ALA), S-adenosylmethionine (SAM-e), selenium and B-complex vitamins (256). The use of antioxidants such as NAC prevents mitochondrial oxidative stress during chronic T-cell stimulation and restores the function of exhausted T cells (136, 257). In addition, replenishment of B-complex vitamins, such as B-12, B-6 and folic acid, is suggested to prevent homocysteine accumulation and to enhance the availability of methyl groups (225, 228, 256)

For the treatment of SIBO outbreaks, as a consequence of immunosuppression and mucosal inflammation, the use of antibiotic agents is considered relevant (258). Also, the potential of antihistamines to reduce inflammation caused by histamine accumulation due to decreased DAO activity is highlighted (259). These drugs block histamine H1 receptors and may influence immune responses, including the reduction of the Th2 response (259, 260). Second-generation antihistamines have less impact on the CNS, which decreases sedation symptoms compared to first-generation antihistamines (260)

During states of inflammation or infection, the immune system may increase its demand for glutamine, especially in activated T cells (261, 262). These cells experience increased glucose and glutamine consumption due to aerobic glycolysis and increased cell proliferation (261, 262). Therefore, it may be beneficial to consider glutamine supplementation. Supplementation with nicotinamide mononucleotide (NMN), as a precursor for NAD biosynthesis, offers the ability to restore depleted NAD levels due to increased PARP-1 activity in both neuronal cells and cells of the immune system (262, 263). Maintaining NAD levels is crucial for preserving the antiviral activities of PARPs (262, 263).

Astragalus, known for its immunomodulatory properties, could help reduce ROS production and improve CD4 T-cell deficient response by strengthening the immune system and increasing the CD4/CD8 T-cell ratio, thus improving its ability to control chronic infection (264, 265). It has also been observed that astragalus can prevent EBV reactivation, so it could be useful in conjunction with antiviral treatment to prevent EBV reactivations (266).

On the other hand, protein supplementation may help to maintain protein synthesis during chronic inflammatory states, in addition to improving the immune response (267), while creatine supplementation could prevent protein degradation (268, 269).

Vitamin D supplementation could help to reduce T-cell hyperactivation, resulting in decreased inflammation (270). In addition, it has been suggested that vitamin D may improve insulin sensitivity, which could have additional benefits in inflammatory diseases and insulin resistance (148).

Melatonin, in addition to improving sleep, may have protective effects on the CNS (271). It has been observed to reduce neuronal death, decrease astrocyte and microglia activation, as well as neuroinflammation (271). In addition, it positively regulates SIRT1 expression, which in turn decreases NF-kB pathway activation and mitigates neuronal mitochondrial dysfunction (271). It also decreases oxidative stress by stimulating antioxidant enzymes and neutralizing ROS (272)

## 8 Implications and conclusions

Among the mechanisms associated with the development of LC and ME/CFS are viral persistence, immunological alteration, the appearance of autoimmune diseases and hormonal imbalances (129, 273–275). Both exhibit CD8 T-cell hyperactivation, T-cell exhaustion, and hypopituitarism (28, 30, 52, 53, 273, 276–279). In addition, decreased levels of DHEA-S in ME/CFS and testosterone in LC have been observed (280–287). In this context, a proposal is presented on the possible sequence of events that could be involved in the development of these diseases, focusing on the interaction between immune hyperactivation, autoimmune hypophysitis or pituitary hypophysitis and immune depletion. It is postulated that infection with SARS-CoV-2 or another virus triggers an uncontrolled immune response in genetically predisposed individuals (HLA-DRB1), with excessive release of proinflammatory cytokines and cellular activation. This cascade of events may lead to initial autoimmune hypophysitis in genetically predisposed patients or to direct damage of the pituitary due to infection or by its inhibition by increased proinflammatory cytokines, resulting in decreased production of pituitary hormones, mainly ACTH. As the disease progresses, the decrease in cortisol and prolonged exposure to antigens may lead to relative immunodeficiency and depletion of the immune system, which is reflected in an increase in the expression of molecules such as PD-1 and CTLA4 (143). It is suggested that this feedback loop between initial hyperactivation, hypophysitis/pituitary hypopituitarism and immune depletion may contribute to the development and persistence of LC and other infectious syndromes such as ME/CFS.

We propose that LC, ME/CFS and post-COVID-19 vaccine syndrome could be included in the ASIA syndrome. Patients with these diseases share the symptoms present in ASIA syndrome, such as chronic fatigue, arthralgias, myalgias, non-refreshing sleep, sleep disturbances, post-exertional malaise, cognitive impairment, memory deficits, inattention, allergies, gastrointestinal symptoms, irritable bowel syndrome, small fiber neuropathy, recurrent urticaria, orthostatic intolerance, and postural tachycardia syndrome (POTS) (74, 129, 273, 288–293). In addition, there are isolated reports confirming this relationship, where some patients have developed ME/CFS after hepatitis B vaccination and meet the criteria for ASIA syndrome (294). It is even suspected that the same occurs in some genetically susceptible individuals after HPV vaccination who develop a chronic dysautonomia syndrome similar to ME/CFS (74, 295). On the other hand, the COVID-19 vaccine, although the specific adjuvants have not been identified, is shown to contain components that can hyperstimulate the immune system, leading to clinical manifestations that meet the minimum criteria for the diagnosis of ASIA syndrome (74, 296, 297). In the case of LC and infectious ME/CFS, adjuvants that could induce hyperactivation and depletion of the immune system by chronic exposure would be viral antigens, interacting with genetically predisposed HLA-II alleles (HLA-DRB1), similar to ASIA syndrome (61).

Polymorphisms in the HLA-DRB1 gene may predispose to an altered immune response, increasing the risk of autoimmune diseases and an exaggerated response of the immune system to certain pathogens or vaccines, as in ASIA syndrome (74, 96, 103, 104). In both classic cases of ASIA syndrome and vaccinations, as well as in LC and ME/CFS, it is foreign antigens that trigger this immune hyperactivation (61, 104). However, the main difference lies in the fact that in most cases of ASIA syndrome, the clinical manifestations and autoimmune diseases developed are usually not chronic and disappear when the adjuvant is removed (64). In contrast, this may not occur in cases of vaccines-mediated or viral persistence in patients with LC and infectious ME/CFS, as the adjuvant (viral antigens) may persist chronically, resulting in the permanence of immunologic alterations and clinical manifestations.

This developmental pattern is compatible with that of other autoimmune diseases caused by viral infections, as in multiple sclerosis (122, 298, 299). Both CD8 and CD4 T cells play a crucial role in the control of EBV viral infection (122, 298, 299). *In vitro* studies have shown that CD8 T cells have a predominant role in killing infected cells, whereas CD4 T cells collaborate with CD8 T cells and can also directly attack infected cells (122, 298, 299). *In vivo* experiments suggest that CD4 T cells contribute to the early control of EBV infection, but their efficacy in restricting viral replication is compromised in the absence of CD8 T cells (298, 299). Further depletion of CD8 T cells along with CD4 T cells leads to an increase in viral titers, indicating that CD4 T cells may play a role in immune control of EBV (298). Specifically, CD4 T cells show lower efficacy in recognizing EBV-transformed B cell lines that expressed HLA-DR15 (121, 122). This may suggest that the main effect appears to be mediated by HLA-II genes, and not by class I genes (122, 299). In addition, there are cross-reactivity with molecular mimicry between EBV and CNS autoantigens that could amplify T-cell and antibody responses (300). Thus, impaired recognition of EBV-infected or latent EBV-infected cells by CD4 T cells in individuals with the HLA-DR15 allele may result in uncontrolled expansion of CD8 T cells and increased production of anti-EBNA-1 antibodies, contributing to infectious mononucleosis and the development of multiple sclerosis (122, 299, 300). In this context, ineffective immune control of EBV-transformed B cells that stimulate autoreactive T-cell responses could facilitate both the initial activation of cross-reactive T cells and their local re-stimulation by EBV-infected B cells, thus promoting autoimmunity in the CNS of patients with multiple sclerosis (300).

The progression of this CD8 T-cell hyperactivation leads, in the long term, to immune exhaustion due to chronic exposure to viral antigens (239, 301). Thus, this process evolves from an initial phase of immune hyperactivation, as seen in infectious mononucleosis, to an exhausted T-cell response, which increases the risk of developing EBV-associated neoplasms (298, 301). This pattern has also been observed in the development of other EBV-associated autoimmune diseases, such as lupus erythematosus (223). In addition, this immune hyperactivation and subsequent T-cell depletion is also observed with other pathogens such as cytomegalovirus, hepatitis B virus, hepatitis C virus, HIV and SARS-CoV-2 (239, 301, 302).

Although depletion of CD8 PD-1+ T cells leads to a decrease in their cytotoxic capacity and impaired control of chronic infections or tumors, this phenotype is thought to be adapted to minimize tissue damage while maintaining a critical level of pathogen control (223, 301, 303). This state limits the risks of cross-reactive tissue injury, but at the same time may explain why EBV replication is not completely suppressed (223). It is thought that PD-1 signaling may attenuate the storm of damaging cytokines during T-cell hyperactivation, thus allowing cytotoxic T-cell functions to effectively control EBV infection and associated tumorigenesis (301). This suggests that CD8 PD-1+ T cells may contribute to the immune control of persistent infections such as EBV, and PD-1 blockade with ICI, may not be beneficial (301). It has been observed that PD-1 blockade during primary infection increases viral loads, generates reactivations of other pathogens, increases tumor burden, and increases elevated production of immunosuppressive cytokines (301). Therefore, the application of ICI could exacerbate viral infection and associated immunopathology, such as autoimmunity (223, 301). This phenomenon is evidenced by increased autoimmune hypophysitis during treatment with ICI (67).

Furthermore, it is important to note that viral infections, such as that caused by EBV, can trigger both CD8 T-cell autoimmune responses and the production of autoantibodies against the adenohypophysis, due to the expression of viral antigens that exhibit molecular similarity to ACTH (243, 304). Thus, not only can an infectious agent better survive in a poorly directed immune response, but if autoantibodies are directed against critical molecules (ACTH), the infection can thrive in a host with a weaker immune system (42).

**Figure 3:**
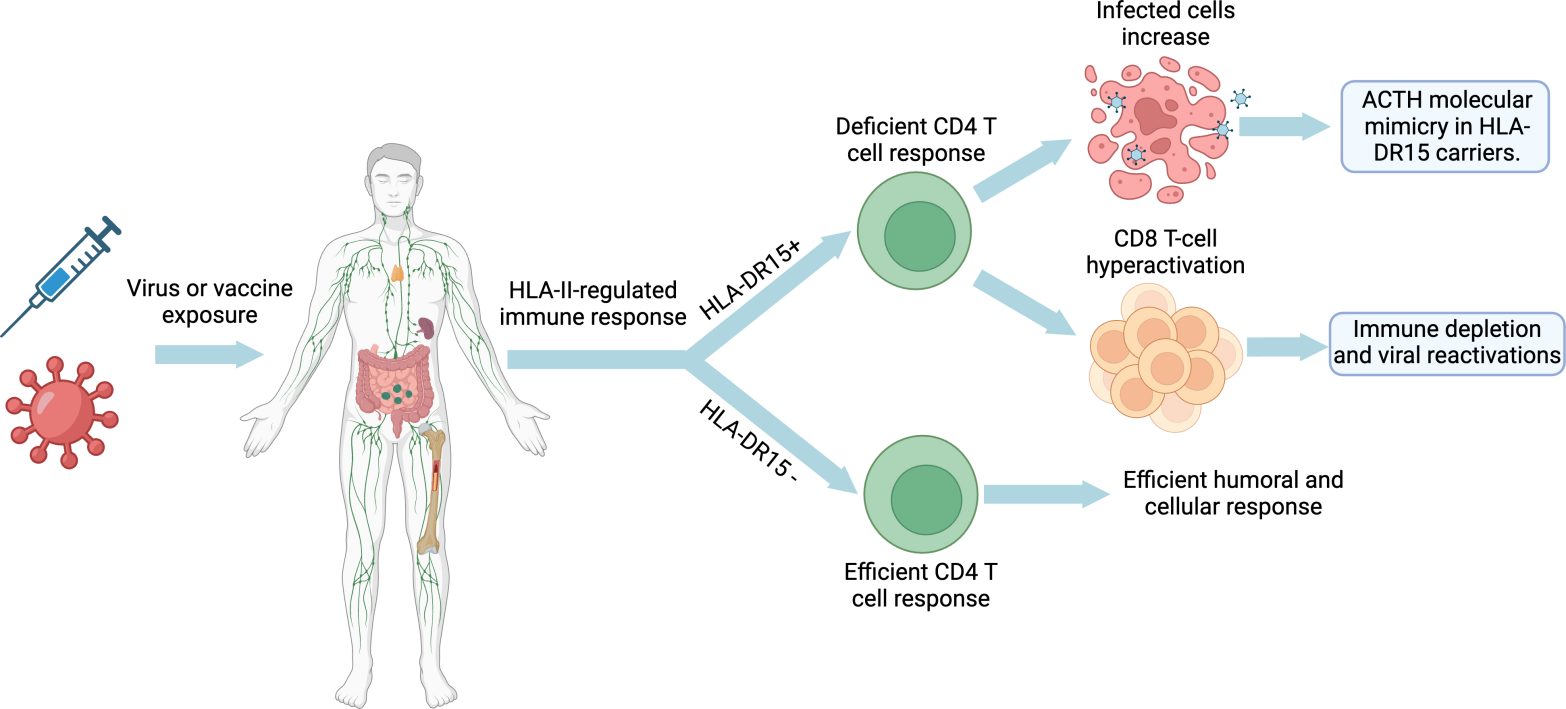
Developmental model of long COVID, ME/CFS and post-COVID-19 vaccine syndrome: Impact of HLA-DR15 on the immune response and molecular mimicry. Created with BioRender.com.

In summary, both ME/CFS caused by EBV or other pathogens, SARS-CoV-2-induced LC, and post-COVID-19 vaccine syndrome could follow a model of autoimmune disease development like that of ASIA syndrome, multiple sclerosis, or lupus erythematosus, but with a pattern of autoimmunity against the adenohypophysis (**Figure 3**). This model implies a complex interaction between immune hyperactivation, autoimmune hypophysitis or pituitary hypofunction, and immune exhaustion, as a consequence of impaired recognition of infected cells by CD4 T cells. It is postulated that viral infection could trigger CD8 T-cell hyperactivation and increased antibody production to compensate for this deficient CD4 T-cell response in genetically predisposed individuals (HLA-DR15), which may lead to autoimmune hypophysitis or direct pituitary damage. As the disease progresses, depletion of certain hormones and prolonged exposure to viral antigens can lead to the exhaustion of the anti-viral immune response. This feedback loop between initial hyperactivation, pituitary dysfunction and immune exhaustion could contribute to the development and persistence of LC and ME/CFS. Furthermore, both diseases by sharing similar symptoms and clinical manifestations to ASIA syndrome should be included in ASIA syndromes.

Thus, the persistent symptoms of LC and ME/CFS are a result of ongoing CD8 T-cell hyperactivation and chronic release of proinflammatory cytokines, exacerbated by viral reactivations due to T-cell exhaustion (180, 273). In addition, this uncontrolled immune response could be potentiated by impaired pituitary function due to autoimmune damage, which manifests in symptoms varying in severity, such as fatigue, orthostatic hypotension and sleep disturbances, depending on the degree of pituitary damage (42, 59). This relationship is reflected in the improvement of symptoms in both diseases with corticosteroid treatment (244, 305–308).

## Author contributions

MR-P: Writing – original draft, Writing – review & editing. AZ: Writing – original draft, Writing – review & editing. BP: Writing – original draft, Writing – review & editing.
